# Brain extracellular levels of dimethylarginines (ADMA, SDMA) and cGMP and their modulation by exogenous S-adenosylmethionine in rats with acute liver failure

**DOI:** 10.1186/2193-1801-4-S1-P8

**Published:** 2015-06-12

**Authors:** Anna Czarnecka, Jan Albrecht, Magdalena Zielińska

**Affiliations:** Department of Neurotoxicology, Mossakowski Medical Research Centre, Polish Academy of Sciences, Poland

**Keywords:** asymmetric dimethylarginine (ADMA), S-adenosylmethionine (SAM), hepatic encephalopathy

Acute liver failure (ALF) instantly evokes symptoms of hepatic encephalopathy (HE), mainly attributed to hyperammonemia. Ammonia neurotoxicity is related to disturbances in the nitric oxide (NO)/cGMP pathway. The methylated derivative of L-arginine (MDALs) - asymmetric dimethylarginine (ADMA) but not symmetrically methylated arginine (SDMA) is an endogenous inhibitor of nitric oxide synthase. Elevated blood and brain ADMA was reported in HE patients and experimental animals. The question arose whether ALF evokes changes in the incorporation of the methyl donor S-adenosylmethionine (SAM) to MDALs, and how they affect cGMP accumulation. To this end, we investigated the effect of intracortical perfusion of SAM on the brain extracellular levels of ADMA, SDMA and cGMP in rats with ALF in the thioacetamide (TAA) model. Sprague Dawley rats (250–270 g) received three i.p. injections of TAA (300 mg/kg) at 24 h intervals. Bilateral microdialysis of the prefrontal cortex was carried out 24 h after the last TAA administration. SAM at 2 mM concentration in artificial cerebrospinal fluid was infused for 40 min and then the medium was changed back. The extracellular levels of ADMA and SDMA were analyzed using positive mode electrospray LC–DMS–MS/MS and cGMP was determined with cGMP enzyme immunoassay. ALF resulted in the increased extracellular levels of ADMA (by ~800%) and SDMA (by ~250%). Moreover, in ALF rats infusion of SAM decreased the ADMA and SDMA (~30%) as compared to the basal value. It seems reasonable that an excessive amount of substrate inhibited the enzymes synthesizing MDALs. On the other hand, the cGMP level did not differ between control and TAA rats. SAM increased by ~90% cGMP only in the control group, what indicates affected NO/cGMP pathway in TAA model. The study demonstrates, that ALF modulates methylation of arginine to MDALs in a manner affecting cGMP accumulation.

